# A List of Cases Discharged from the Surgical Wards of the Pennsylvania Hospital, during the Month of April, 1843

**Published:** 1843-05-27

**Authors:** Ellerslie Wallace

**Affiliations:** Resident Surgeon


### CLINICAL LECTURES AND REPORTS.

### A LIST OF CASES

### DISCHARGED FROM THE SURGICAL WARDS OF THE PENNSYLVANIA HOSPITAL,

*During the month of April*, 1843.

[Reported by Ellerslie Wallace, M. D., Resident Surgeon.]

One case of injury from machinery, all the toes of the right foot being torn off. Under treatment 113 days.

Discharged, cured, the stump being smooth and healthy.

Two cases of ophthalmia. Under treatment sixty-four days.

Discharged, cured.

Two cases of paronychia. One under treatment fifty-four days, the other, thirty days.

Discharged, cured.

One case of onychia. Under treatment twelve days.

Discharged, cured.

Six cases of contusion, in four of which the injury was trifling, and the patients were discharged, cured, in from two to nine days. One case occurred in an old man, of bad constitution; severe contusion of the left knee. He was under treatment seventy-two days, and then was discharged, cured. One case, in a young man who had fallen prone, and whose loins had been passed over by a loaded cart. Admitted twenty-two hours after the accident. No fracture could be discovered, and motion and sensation in lower extremities perfect. His pain was very great, and any motion of the pelvis increased it. Abdomen hard, and very tender on pressure; tympanitis slight. He had had repeated vomitings for the last few hours, which still continued. Neither urine nor fæces had been passed since the injury. Pulse 88, and feeble. After the catheter had been passed an enema was given, with the effect of opening his bowels freely, and cut cups were applied to lumbar regions and spine. A large emollient poultice was applied to the abdomen, and a sinapism to the epigastrium. Ice in small pieces was ordered, to allay his thirst, which was intense. No medicine could be retained by the stomach for a sufficient length of time to be of any service. Under the treatment he was more comfortable after the lapse of an hour, with less frequent vomiting, and less soreness on pressure. During the night he passed his urine four times, and in the morning appeared a little better. At 8 o'clock, A. M., the vomiting became more frequent, the thirst also increased; and at 9 o'clock, eighteen hours after his admission, he was seized with symptoms of peritonitis. The treatment now instituted consisted chiefly of full venesection, followed by an enema and hot stupes to abdomen. For a short time his pain was relieved, but it soon returned with increased violence; and at 5 o'clock, P. M., twenty-six hours after his entrance into the hospital, he died. An autopsy was asked for, but refused.

One case of sprained ankle, caused by a fall down the hold of a vessel. The constitutional treatment instituted was antiphlogistic, and the local treatment adopted was rest, elevation, and cold lead water poultices applied to the part for several days. Afterwards a soap plaster and bandage were used, and after thirty days he was discharged at his own request, nearly well.

One case of rupture of external lateral ligament of the knee. Under treatment 105 days.

Discharged, cured.

One case of fracture of the os humeri in its lower third. Treated by a bandage from the ends of the fingers up to the axilla, and an angular splint on the inner side of the upper extremity, extending from the axilla to the ends of the fingers; and a short straight splint on front, back, and outside of the arm. Under treatment thirty-two days.

Discharged, cured.

One case of fracture of the olecranon process, without much displacement. Treated by a splint applied to the front of the upper extremity, keeping it in the extended position, with a bandage from the ends of the fingers up to the axilla, having a figure of 8 turn at the elbow to retain the olecranon in place. Under treatment forty-five days.

Discharged, cured.

Three cases of fracture of the lower third of the radius. Treated by a splint on front and back of the forearm, extending to the ends of the fingers, with compresses to retain the fragments in apposition. In from forty-two to fifty-nine days all were

Discharged, cured.

One case of fracture of the patella in a lad seventeen years old, caused by muscular contraction. Separation one and a half inches. Treated by a straight splint to back of lower extremity, keeping it in the extended position, with a bandage from the foot up to the groin, having a figure 8 turn at the knee to keep the fragments in apposition.

Under treatment eighty-nine days. Discharged, with a ligamentous union one-third of an inch long. Motions of flexion and extension of leg increasing slowly.

One case of fracture of the leg. Fibula broken near the upper end, and both bones broken in the middle third, caused by a fall of coal in a mine at Pottsville, Schuylkill county, Pennsylvania. The fracture was reduced soon after the accident, and he was sent by railroad to Philadelphia; the leg being secured by splints and a bandage. Upon the removal of the dressings the soft parts were found to be severely contused, and the swelling and irritation were very great. Violent inflammation followed, with the formation of an extensive abscess, by which the fracture became compound. The constitutional symptoms were very severe, requiring careful attention and support. The leg was treated in a fracture box, and after being under treatment ninety-four days, he was

Discharged, cured.

One case of compound fracture of the skull, with depression, caused by a fall from a horse. When admitted, the man was labouring under symptoms of concussion of the brain, but could answer questions satisfactorily, and assisted in taking off his clothes. He had been bleeding from the right nostril since the injury, but the hemorrhage had ceased before he was brought in. There was no hemorrhage from the ears. The left eye was somewhat prominent, and the lids largely ecchymosed; but no contusion could be discovered near the eye. The fracture was over the coronal suture, midway between the sagittal and squamous sutures, about an inch long, and the depression slight. The wound of the scalp was somewhat less than an inch in length. His hands and feet were cold; his pulse 50, and rather feeble. Sinapisms were applied to his ankles and feet, and over the abdomen, and in less than an hour reaction came on violently, with well marked symptoms of compression; strong spasms, and face almost livid; the blood was also flowing from the nostril; his pulse was 52, and full and strong, and his respiration loudly stertorous. He was freely depleted, by which the spasms abated; his face became more natural, and also his respiration. His pulse rose to 58, and became much softer. The relief, however, was but temporary; the unfavourable symptoms soon returned, and in three hours from the time of his admission he died. An autopsy could not be obtained; but it is probable that a greater injury existed than the one above mentioned. The symptoms were very similar to those observed in a case which occurred in this city about a year since, in which a post-mortem examination showed an extensive fracture of the base of the skull.

One case of caries of the spine and bones of the pelvis and lower extremity, with extensive tuberculosis of the lungs. Cavities had been formed in the upper lobes of both lungs. There was also found a psoas abscess, with a large quantity of pus and cheesey matter under the muscle, and a large cold abscess on the outside of the thigh, communicating with the hip-joint. Tubercles of considerable size were also found on the bones. The patient was a man about twenty-seven years old, and had been in the Hospital but three days at the time of his death.

One case of erysipelatous inflammation of the arm, following a cut of trifling size, received four days previous to his entrance. The arm was swollen and very painful, and the patient had some fever. He was put in bed; purged with Pil. Hydrarg. and Sulph. Magnesiæ; ordered a low diet; and lint, wet with cold mucilage, was laid over the arm. After being under treatment two days, he was, at his own request,

Discharged, much better.

One case of axillary abscess, extending far under the great pectoral muscle. Opened four days after his admission. Under treatment fifteen days.

Discharged, cured.

One case of inflamed face. Under treatment ten days.

Discharged, cured.

One case of furunculus. Under treatment twelve days.

Discharged, cured.

One case of ulcer of the leg, resulting from contusion on the spine of the tibia. Under treatment thirty days.

Discharged, cured.

One case of ganglion on the dorsum of the foot. Treated by punctures and compression. Under treatment forty-four days.

Discharged, cured.

—

From the venereal wards there were discharged *four cases*, one of which had been under treatment for a large cluster of venereal warts. By the knife, and repeated applications of the pure nitric acid, they were entirely removed; some thickening of the prepuce remaining, the part was rubbed with ung. hydrarg. campħ., with the effect of producing absorption of the lymph, and he was

Discharged, cured.

—

Besides the above, the following cases were treated, and went out, by their own request, immediately.

Five cases of incised and lacerated wounds of the scalp.

Three cases of incised wounds of the upper extremity; and

One case of a woman who had swallowed a large pin. The head could be felt by the end of the finger, and the pin had penetrated the posterior part of the larynx. It was removed by a pair of polypus forceps.

